# Growth, lifetime, directional movement and myosin-dependent motility of mutant keratin granules in cultured cells

**DOI:** 10.1038/s41598-021-81542-8

**Published:** 2021-01-27

**Authors:** S. M. Lehmann, R. E. Leube, R. Windoffer

**Affiliations:** Institute of Molecular and Cellular Anatomy, RWTH Aachen University, Wendlingweg 2, 52074 Aachen, Germany

**Keywords:** Cell biology, Fluorescence imaging, Time-lapse imaging

## Abstract

Intermediate filament polypeptides (IFPs) are prominent components of cytoplasmic aggregates, which are pathognomonic for multiple diseases. Recent observations in cultured cells suggest that they are dynamic and subject to regulated turnover. The emerging concept is that multiple factors contribute to motility and turnover of IFP-containing aggregates. To understand their relative contribution, quantitative tools are needed. The current study addresses this need using epithelial cells producing mutant keratin IFPs that have been identified as the cause of the hereditary blister-forming skin disease *epidermolysis bullosa simplex*. Digital image analysis of individual granules allowed mapping of their complete life cycle, with information on multiple characteristics at any given time-point. The deduced signet features revealed rapid granule fusion and directed transport from the periphery towards the cell centre, and a limited, ~ 30 min lifetime with a slow, continuous growth phase followed by fast disassembly. As paradigmatic proof-of-principle, we demonstrate that inhibition of myosin II selectively reduces granule movement, linking keratin granule motility to retrograde cortical acto-myosin flow. The newly developed methods and established parameters will help in the characterization of known and the identification of novel regulators of IFP-containing aggregates.

## Introduction

Cytoplasmic protein aggregates are hallmarks of many diseases, which affect various organs and lead to diverse pathologies such as neurodegeneration, myopathy or epidermal blistering. The protein composition of these aggregates varies considerably but intermediate filament polypeptides (IFPs) have been shown to be involved in most instances^1–6^. Whether and how the different cell type-specific protein aggregates contribute to the respective pathogenic mechanisms still remains to be elucidated. The initial view that IFP-containing aggregates are simply static cellular debris has been increasingly questioned in the last decades and various studies support the view that they are quite dynamic^7–10^. This has kindled interest in understanding and modulating the dynamic processes of protein aggregate formation in order to interfere with disease initiation and progression for the benefit of affected patients.

*Epidermolysis bullosa simplex* (EBS) is a prototypic protein aggregate-forming disease, which affects the epidermis. The autosomal-dominant disease is caused by mutations in keratin 5 and keratin 14. It manifests with severe blistering in response to mild mechanical trauma because of cytolysis in the basal cell layer of the epidermis^3^. At the subcellular level, EBS is characterized by disruption of the keratin filament network and occurrence of keratin-positive cytoplasmic aggregates^3,11,12^. Overexpressing EBS-mutant human keratin 14 in mice phenocopies the EBS phenotype^13,14^. Similarly, expression of EBS-mutant keratins in cultured cells leads to the formation of spherical keratin-containing cytoplasmic granules. These culture systems have helped to characterize modifiers of keratin granule formation including signalling molecules, heat shock proteins, ubiquitin ligases, actin filaments and adhesion sites^9,10,15–19^. The emerging concept is that there is not a single factor responsible for keratin aggregate formation, turnover and motility but that multiple factors contribute in different ways and to different degrees. The elucidation of this multifactorial scenario requires sensitive means to qualitatively and quantitatively assess the contribution of each factor to keratin granule dynamics. We therefore developed imaging and analysis routines to characterize the dynamic behaviour of mutant keratin granules. We demonstrate that they can be used to precisely describe at high temporal and spatial resolution the assembly, growth, disassembly, lifetime and motility of single keratin granules. As proof-of-principle, we quantify, for the first time, the inhibitory effect of the actin motor protein myosin II on the speed and persistence of motion of keratin granules.

## Results

### Automated image analysis allows mapping of the appearance, motility and disappearance of mutant keratin granules

**Figure 1:**
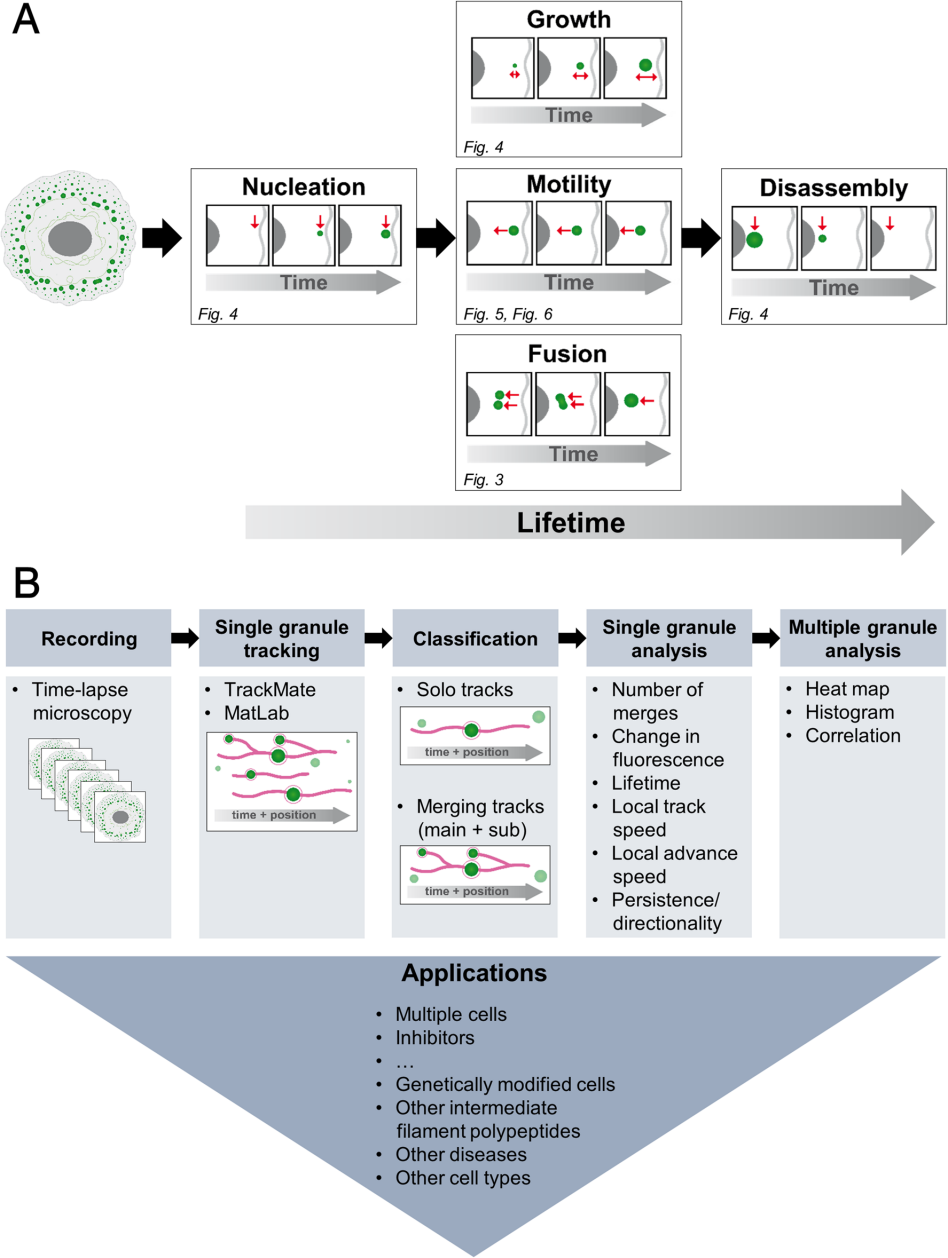
The schemes show overviews of the examined properties of mutant keratin granules and the workflow to determine them in quantitative terms. (**A**) The scheme depicts a typical EYFP-K14R_125_C-producing MCF-7 cell at the left and the different events which were studied at the single granule level with reference to the figures describing the respective analyses. They comprise nucleation, growth, motility, fusion and disassembly of keratin granules. (**B**) The scheme summarizes the different analysis steps of keratin granule dynamics beginning with the recording of changing fluorescence patterns in living cells producing fluorescence-tagged EYFP-K14R_125_C, followed by tracking of single granules, classification of tracks and determination of different granule properties at the single granule level and final multimodal statistical evaluation of entire granule cohorts in a given cell. The manuscript further describes how these methods can be used for the analysis of granule dynamics in different cells and its response to specific drugs. The described approach can be extended to examine keratin granule dynamics in genetically modified cells and can be extrapolated to other intermediate filament polypeptides, diseases and cell types.

The scheme in Fig. 1 serves as a guide for the approaches and results described in this paper. The previously described mammary adenocarcinoma-derived MCF-7 cell line producing fluorescent protein-tagged keratin 14 mutant EYFP-K14R_125_C^9^ was selected to establish methods for measuring the dynamic properties of EBS-mutant keratin aggregates. K14R_125_C is the most frequently mutated residue in EBS (www.interfil.org).

**Figure 2:**
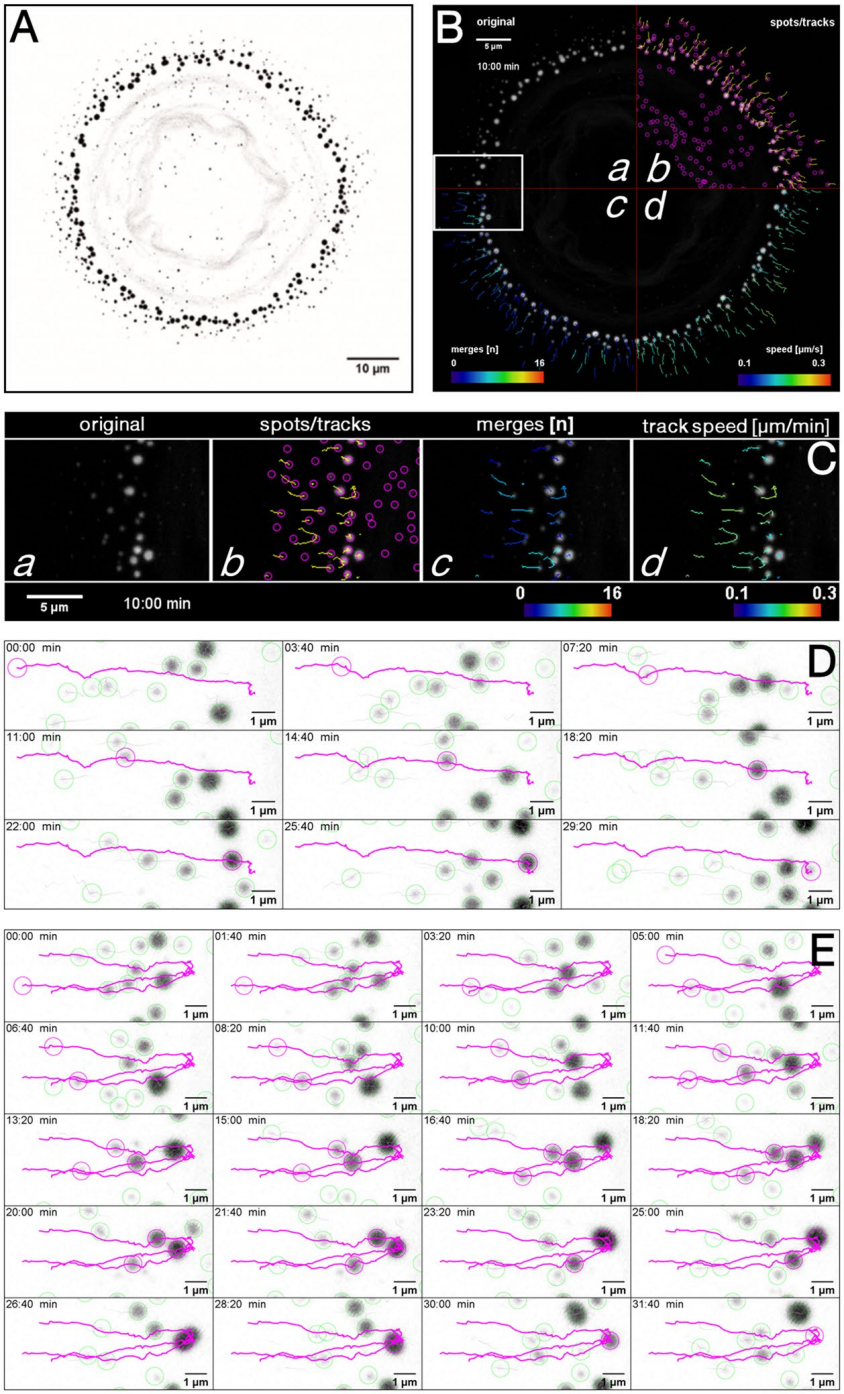
Image analysis of fluorescence recordings in MCF7 cells producing yellow fluorescent protein-tagged keratin 14 mutant EYFP-K14R_125_C provides quantitative information on keratin granule motility and fusion. (**A**) The fluorescence microscopy (inverse presentation) is taken from Movie 1 and corresponds to the first image taken at time point 0 min. (**B**) The composite fluorescence micrograph is taken from Movie 2. The four quadrants depict different stages of image documentation and analysis. (a) presents the projected fluorescence recording at time point 10 min. The nuclear region is in the center and the granules are restricted to the cell periphery increasing in size towards the cell interior. (b) Detected granules are circled (magenta) and corresponding tracks are shown as yellow lines. Only the track paths of the last ten frames are shown. Note that the very small and weakly fluorescent granules, which are detected in the cell center, lack a track because of their short lifetime. (c) depicts the color-coded number of fusion events observed for each track. (d) shows the color-coded mean speed for each track. (**C**) The region boxed in white in B is shown at higher magnification with the full sequence provided as Movie 2. (**D**) Depicts a representative track of a single, non-merging keratin granule (surrounded by magenta circle) that was followed during its entire lifetime of 29 min 20 s moving from the cell periphery at left towards the cell center. For better visualization other nearby granules are circled in green without their corresponding tracks. The entire sequence is shown in Movie 3. (**E**) Presents tracks of three fusing granules (circled in magenta) during their entire lifetime of 31 min 40 s (cell periphery at left). Other nearby granules are encircled in green without their tracks shown. Corresponding Movie 4 shows the entire sequence.

Figure 2A depicts EYFP-K14R_125_C granules in a round-shaped, vital MCF-7 cell. The fluorescence micrograph confirms previous reports^9,10^ detecting keratin granules in the cell periphery and a residual network that is limited to the perinuclear region. Time-lapse fluorescence recording (Movie 1) revealed that nascent keratin granules appear at different positions close to the cell boundary. The growing granules move towards the cell centre and disappear at a distinct transition zone, which is approximately 10 µm away from the cell boundary and coincides with the border between the peripheral, thin lamellum and the inner, much thicker cytoplasm.

The different steps of subsequent image analysis are depicted in Fig. 2B,C (corresponding Movie 2). Starting with the raw data (Fig. 2Ba,Ca) individual keratin granules were identified by the Laplacian of Gaussian' detection implemented in Fiji's TrackMate plugin (encircled in purple in Fig. 2Bb,Cb). In addition to the peripheral granules, other, less fluorescent granules were also detected throughout the cytoplasm (blinking spots in Movie 1). The tracking algorithm, however, discarded them because of their short lifetime. On the other hand, the movement of the more fluorescent and enlarging peripheral granules could be reliably documented. The yellow tracks in Fig. 2Bb,Cb delineate the movement of single granules that could be followed for 10 or more consecutive frames. Granules frequently fused with each other, resulting in merging of tracks. Figure 2Bc,Cc show colour-coded representations of the number of merging events per track superimposed on the fluorescence recording at time point 10 min. To visualize the speed of granule movement, the displacement rates of entire tracks were determined and are represented quantitatively in colour code in Fig. 2Bd,Cd.

Figure 2D,E further highlight the quality of the image analyses by showing complete tracks of single keratin granules from their first detection to their dissolution. Figure 2D (corresponding Movie 3) depicts a granule that did not fuse with other granules during its ~ 29 min lifetime. We will refer to these track types as "solo" tracks (Supplementary Fig. S1A). The track selected in Fig. 2E (corresponding Movie 4) represents the movement of three granules that fused with each other. This track type is referred to as a "merging" track consisting of a "main" track, which is the longest, in this instance ~ 32 min track, and two shorter "sub tracks", which were ~ 6 min and ~ 18 min, respectively, in the selected example (Supplementary Fig. S1A).

Taken together, the established conditions and methods provide measurable information on the position, movement, fluorescence and fusion frequency of mutant keratin granules and the properties derived from them for entire vital cells at single granule resolution.

### Keratin granule fusion occurs randomly but fusion rates correlate with granule size and density

**Figure 3:**
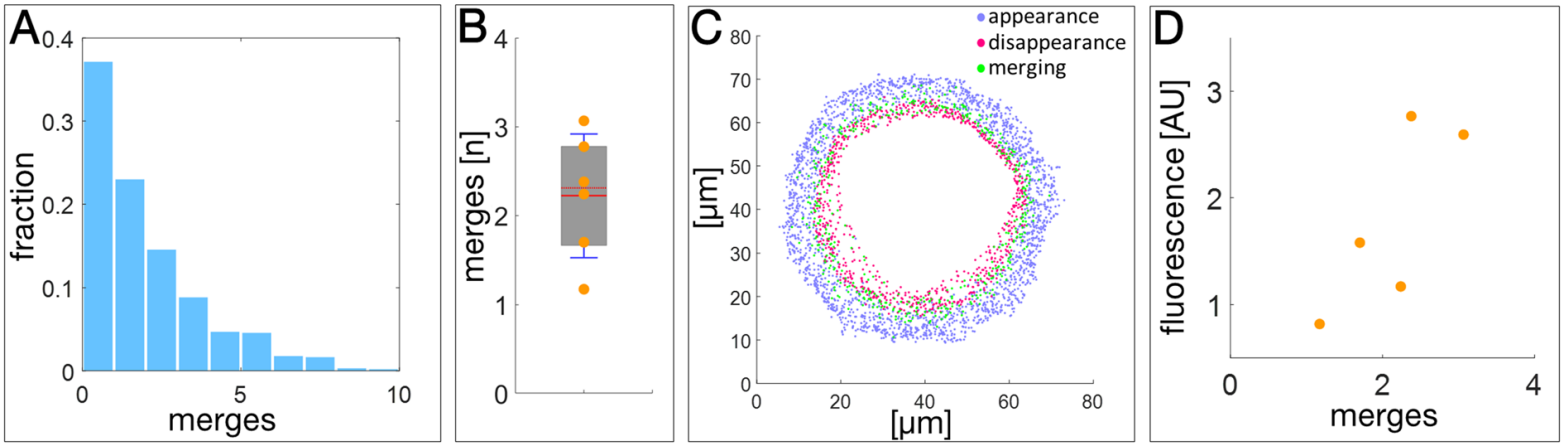
Image analysis of time-lapse fluorescence recordings in EYFP-K14R_125_C-producing MCF-7 cells reveals frequent merging of keratin granules during their lifetime. (**A**) The histogram depicts the number of merging events for each newly formed granule. 820 tracks of moving granules were taken from Movie 1 without any selection filter. (**B**) The whisker box plot shows the number of merging events observed in complete tracks from first detection (appearance) to last detection (disappearance) in each of 6 fluorescence time-lapse recordings. (**C**) The picture illustrates the positions of appearance (blue dots), merging (green dots) and disappearance (red dots) of keratin granules as determined for Movie 1. (**D**) The graph shows the number of merging events versus fluorescence (in arbitrary units, AU) determined from five independent time-lapse fluorescence recordings.

We first wanted to find out, whether position, size or density of keratin granules affect their fusion rate. 62.68% of the 820 granule tracks deduced from Movie 1 were of the merging type. The number of fusions ranged between 1 and 10 (Fig. 3A). Combining track analyses from 6 recordings of 6 different cells showed that fusion occurred 2.22 ± 0.69-times per track (Fig. 3B). Mapping of the merging events revealed that they occur mainly in the middle and inner granule-bearing region (Fig. 3C). Cells with a high overall fluorescence, reflecting a large number of large granules, had a high rate of granule fusion (Fig. 3D). The latter observation may be due to a higher probability of granule approximation.

### Mutant keratin granules have a limited lifetime encompassing a slow growth and rapid dissolution phase

**Figure 4:**
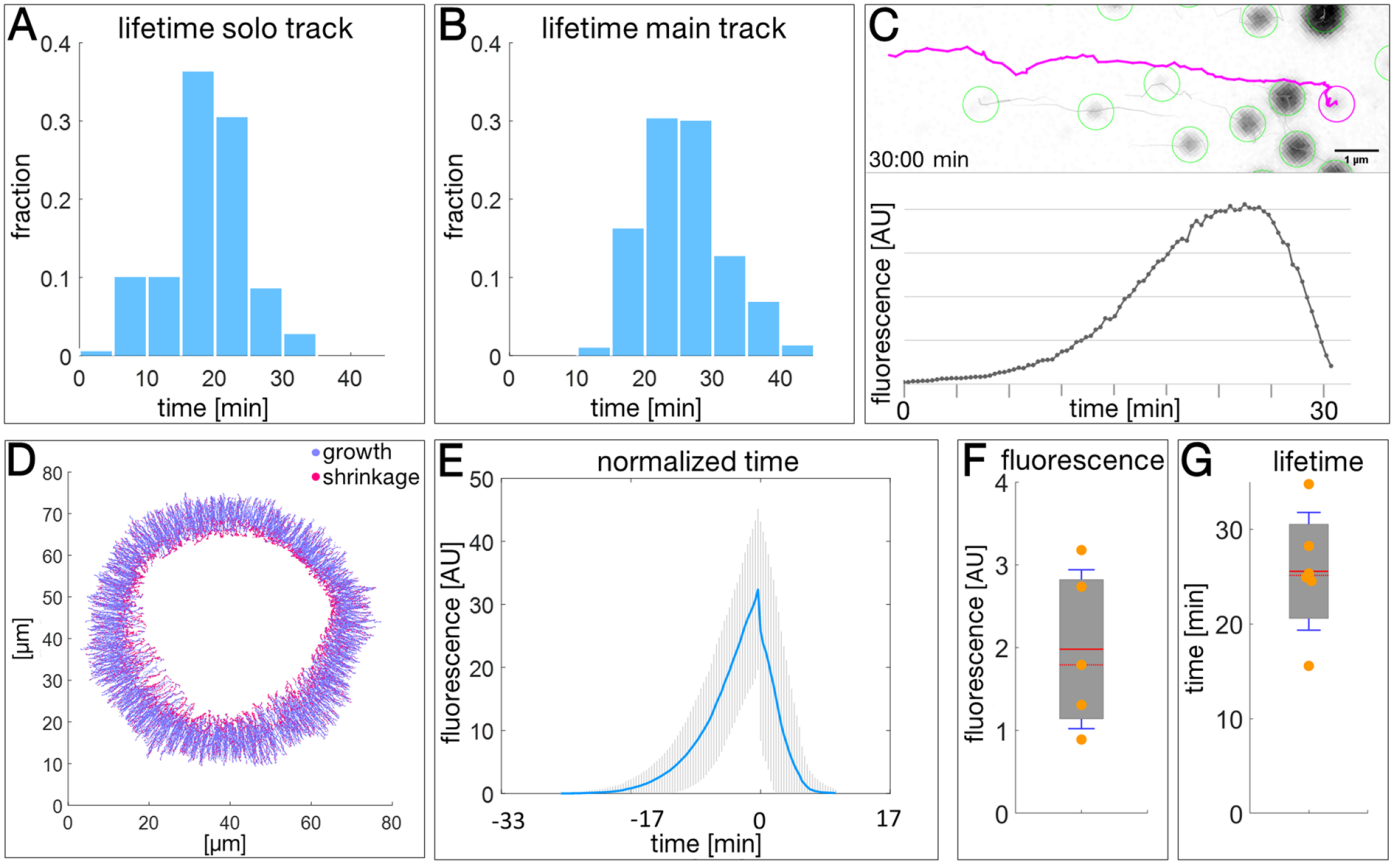
Unique signet features of keratin granule lifetime, growth and disassembly can be deduced from time-lapse fluorescence recordings in EYFP-K14R_125_C-producing MCF-7 cells. (**A**,**B**) The histograms show the lifetime of individual keratin granules, which either did not fuse with other granules (**A**; n = 137) or fused with other granules (**B**; n = 341). For the fusing granules only the longest tracks (main tracks) were analysed (see Supplementary Fig. S1A). The data are taken from Movie 1. (**C**) The photomontage shows the track of a typical single keratin granule projected onto the fluorescence image (inverse presentation) taken at 30 min of Movie 5 and the corresponding fluorescence changes over time in the graph below. Other automatically tracked and segmented nearby granules are encircled in green. (**D**) Illustrates the growth and shrinkage of keratin granules. The change of fluorescence was determined for each individual granule track between each time point. Granule tracks increasing in fluorescence brightness are shown in blue, granules with decreasing fluorescence in red. Data are from the same 820 tracks of Movie 1 analysed in (Fig. 3C). (**E**) The graph shows the relative changes in granule fluorescence over time determined for non-fusing keratin granules (n = 137). The time point of reaching the maximum fluorescence as was set to 0 min for each granule for better comparison. The mean of the resulting fluorescence is depicted in blue together with the corresponding standard deviation from the mean (SD) in grey. (**F**,**G**) The whisker box plots depict the mean maximum fluorescence (**F**) and the mean lifetime (**G**) of keratin granules determined in time-lapse recordings of 5 or 6 cells, respectively. Only complete tracks were analysed.

To find out, whether fusion affected the lifetime of keratin granules, the duration of solo and main merging tracks was compared (Fig. 4A,B). The average lifetime of non-fusing granules turned out to be shorter (18.49 ± 5.92 min) than that of fusing granules (25.82 ± 6.34 min). To understand granule growth kinetics in the absence of fusion, granule fluorescence was examined in non-merging tracks. Movie 5 and Fig. 4C present a prototypical track and the corresponding changes in fluorescence of a single keratin granule. As to be expected, the increase in granule fluorescence corresponded well with the size of the growing granule (Movie 5). Changing fluorescence was therefore taken as a measure of granule growth. The slow initial growth of the nascent granule was followed by an exponential growth phase that levelled off at a distinct maximum. The ensuing disassembly of the granule occurred rapidly with almost linear kinetics. Plotting the positions of initial granule detection together with the positions of last granule detection showed that both events are spatially separated (Fig. 3C). Granule appearance was restricted to the peripheral 2/3 of the granule-bearing zone, granule disappearance to the inner 1/3. Comparing growth and dissolution of granules further demonstrated that granule growth was limited to the peripheral part of the granule-containing zone whereas shrinking occurred only in the inner part of that zone (Fig. 4D).

To see whether the change in fluorescence observed for the selected single granule was representative, normalized granule fluorescence was compared for 137 solo tracks (Fig. 4E). The average granule fluorescence determined for 5 cells, that were imaged with identical settings, varied by a factor of 3. A slow growth phase and a much faster dissolution phase of keratin granules, however, were readily apparent independent of the average fluorescence of granules, whose average lifetime was 25.56 ± 6.22 min (Fig. 4F,G).

### Mutant keratin granules move with decreasing speed as they grow

**Figure 5:**
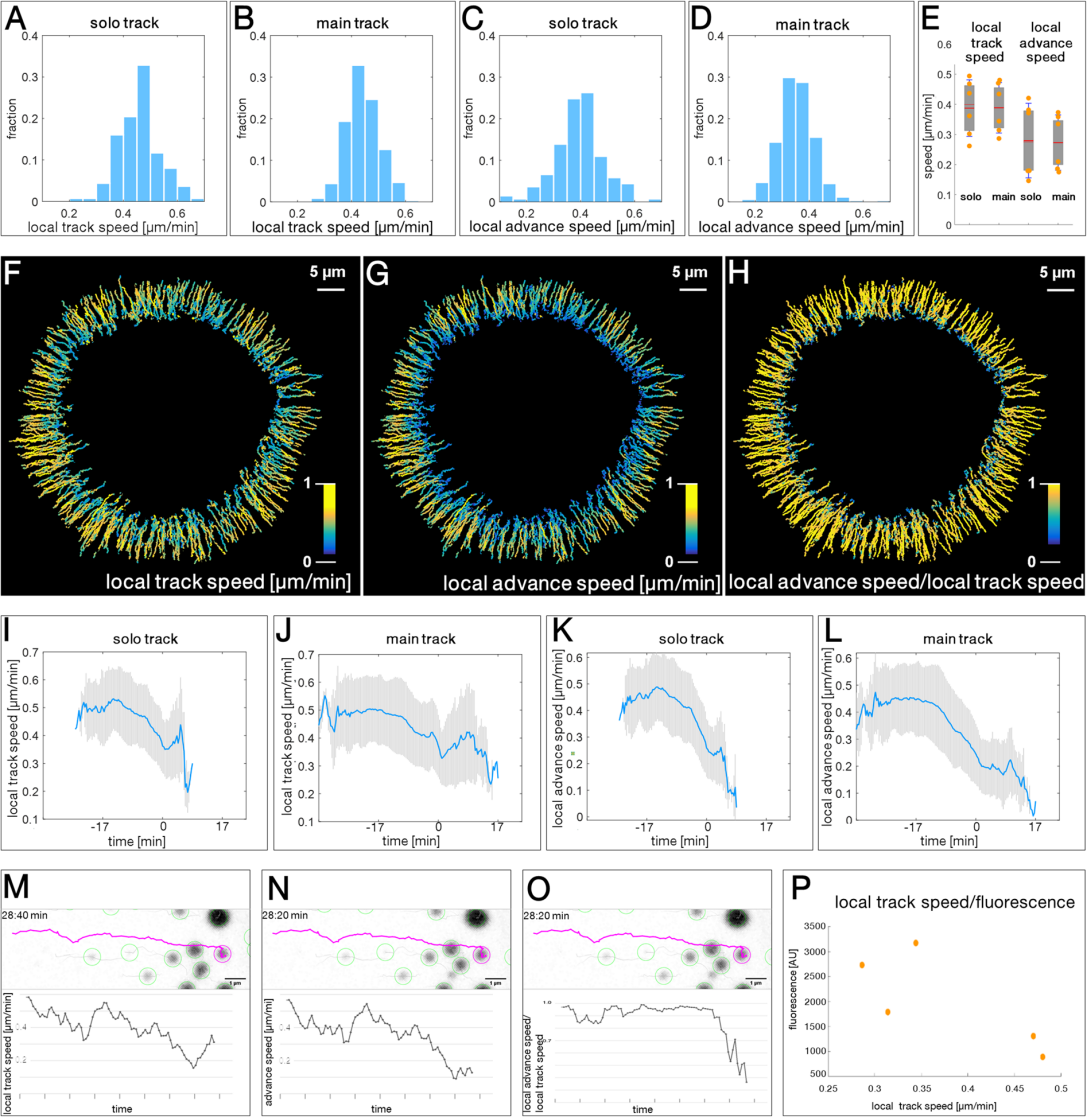
Analysis of keratin granule motility in EYFP-K14R_125_C-producing MCF-7 cells reveals coordinated motility profiles. (**A**–**D**) The histograms depict the local track speed of non-fusing (**A**; n = 137) and fusing keratin granules (**B**; n = 341) and the corresponding advance speed (**C**,**D**). Data are taken from Movie 1. The local track speed is defined for each position as the displacement (µm) per min for an 8 frame window at a given time point and amounts to 0.46 ± 0.06 µm/min for solo tracks (**A**) and 0.44 ± 0.08 µm/min for main tracks (**B**). The advance speed is defined as the shortest distance travelled for an 8 frame window at a given time point. The value for solo tracks is 0.35 ± 0.07 µm/min and 0.39 ± 0.09 µm/min for main tracks. (**E**) The whisker box plot shows local track speed and advance speed for 6 time-lapse fluorescence recordings. Mean speed of solo tracks amounts to 0.39 ± 0.09 µm/min, and 0.39 ± 0.08 µm/min for main tracks. Advance speed of solo tracks is 0.28 ± 0.12 µm/min, and 0.27 ± 0.09 µm/min for main tracks. (**F**–**H**) The color-coded tracks show the local track speed (**F**), advance speed (**G**) and ratios of local advance and local track speed of individual keratin granules ((**H**); corresponding Movie 1) highlighting directed movement toward the cell interior during most of the granule's lifetime and erratic movement just prior to granule disappearance. (**I**–**L**) The graphs show the changes in local track and advance speed for non-fusing (solo track) and fusing (main track) single keratin granules. Data from Movie 1 were used. Curves are normalized with respect to time by setting time point 0 min at maximum granule fluorescence. Blue lines: mean; grey bars: SDs. (**M**–**O**) Tracks of a single, non-merging granule with diagrams showing local track speed (**M**), local advance speed (**N**) and the ratios of local advance speed/ local track speed (**O**) over time. The corresponding animations are provided as Movie 6. The changes of fluorescence over time of this granule are shown in Fig. 4C. (**P**) The graph shows the ratio of local track speed and granule fluorescence for 5 time-lapse fluorescence recordings. Note that increased granule fluorescence correlates with reduced speed.

To gain detailed information on the mobility of keratin granules, the local speed and direction of keratin movement was analysed next. Using data from Movie 1, we determined the local track speed as the distance travelled per time along the track in µm/min for an 8 frame window at any given time point (Supplementary Fig. S1B). The average local track speed of granules along solo tracks was very similar to that of granules moving along main merging tracks (0.46 ± 0.06 µm/min vs. 0.44 ± 0.08 µm/min; Fig. 5A,B). To obtain a measure for the persistence of movement, we determined the local advance speed, which is defined as the speed determined for the shortest distance between the first and last positon of each 8 frame window (Supplementary Fig. S1B). As expected, the values were lower than those for the local track speed but, again, did not differ between solo and main tracks (0.35 ± 0.07 µm/min vs. 0.39 ± 0.09 µm/min; Fig. 5C,D).

To determine whether the findings were representative, we examined the track and advance speed of granules in 6 cells (Fig. 5E). The overall values (mean local track speed for solo tracks: 0.39 ± 0.09 µm/min; mean local track speed for main tracks: 0.39 ± 0.08 µm/min; mean local advance speed for solo tracks: 0.28 ± 0.12 µm/min; mean local advance speed for main tracks: 0.27 ± 0.09 µm/min) were slightly different from the single cell data. Closer inspection revealed considerable heterogeneity and indicated the presence of a group with fast- and another group with slow-moving granules.

Figure 5F,G depicts colour-coded tracks representing local track speed and local advance speed of main tracks. Both parameters are high during most of the granule's lifetime and slow down considerably at the end. The side-by-side comparison further suggested that the local advance speed rapidly slowed down at the end of granule lifetime and slightly before the local track speed slowed down. In accordance, the ratios of local advance speed to local track speed were stable for most of the granule's lifetime and decreased at the end of its lifetime (Fig. 5H). The phenomenon was also apparent in graphs, in which the fluorescence of granules was normalized to the time point of the highest fluorescence level (Fig. 5I–L). The decrease in granule fluorescence (after time point 0 min) was coincident with a transient increase in local track speed. At the same time the local advance speed was drastically reduced. The graphs in Fig. 5I–L further demonstrate that both local track and local advance speed decreased substantially as granules grew (before time point 0 min).

Together, the observations indicated that mutant keratin granules move directionally during most of their lifetime but lose directionality while still remaining motile just prior to dissolution. Support for this idea was also derived from inspection of single tracks at high resolution, revealing erratic granule movement at the end of granule lifetime (Movies 3, 4). The selected track in Fig. 5M–O (Movie 6) exemplifies the described alterations in local track and advance speed and their changing ratios at the end of granule lifetime.

To show that the relationship between granule speed and granule size is a general property, we determined the ratios between local track speed and granule fluorescence in time-lapse recordings of 5 different cells (Fig. 5P). The results show that increased granule fluorescence correlates with reduced velocity, indicating that granule size and speed are inversely related.

### Mutant keratin granules move by directed transport

**Figure 6:**
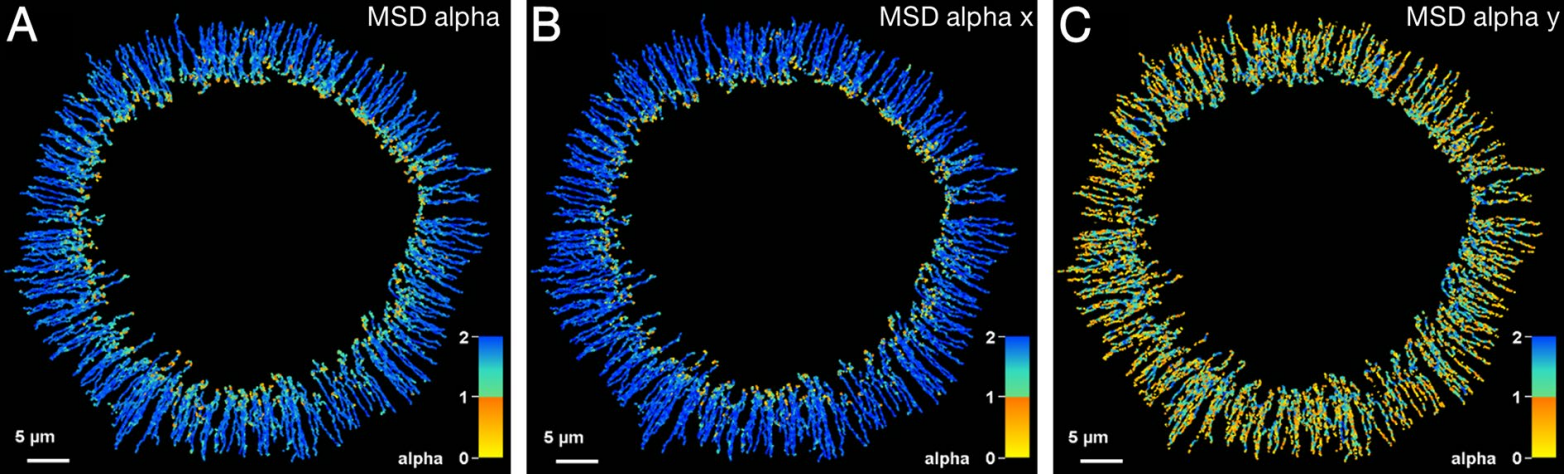
Mean square displacement analyses of keratin granules in EYFP-K14R_125_C-producing MCF-7 cells reveal uniform inward-transport in a restricted region of the peripheral cytoplasm. (**A**) The color-coded tracks depicting the overall alpha value. (**B**,**C**) The color-coded tracks depict the alpha values in x and y, respectively. The x-axis is defined as the axis of transport defined by the start- and end-point of each individual track. Alpha > 1 indicates directed transport, alpha = 0 free Brownian motion, and alpha < 1 restricted motion. Note directed movement along the x-axis and lack of mobility perpendicular to that direction. The end of directed transport along x is indicated by alpha < 1 at the end of the lifetime of the granules. The movement along the y axis (= perpendicular to x-axis) remains random throughout the entire granule lifetime.

The above analyses of local track and advance speed indicated that keratin granules move coordinately toward the cell centre. To distinguish between directed, free and restricted mode of mutant keratin granule transport, we performed mean square displacement (MSD) analyses. The coefficient derived therefrom is the alpha (α) value, which indicates free diffusion if it equals 1, whereas a value of α > 1 indicates movement by active transport, and a value of α < 1 points to motion constrained in space^20^. Analysing the data of Movie 1, the alpha value was determined for each time point of each granule and was projected onto the track data. Figure 6A shows that the tracks display alpha values > 1 almost over their entire length, indicating directed transport. Shortly before the tracks end, values of < 1 indicated the end of directed transport and restricted diffusion for the last frames instead.

To distinguish between inward movement and movement perpendicular to this axis, the movement along the x-axis (defined as the axis between start- and end-point of this track) and y-axis (perpendicular to the x-axis) were analysed separately (Fig. 6B,C). Movement along the x-axis showed alpha values above 1 for most of the granule lifetime, which became only less than 1 at the end. In contrast, the movement along the y-axis showed alpha values below 1 during most of the granule lifetime.

Together, we concluded that directed movement of granules occurs only in one direction, i.e. centripetally towards the cell interior. The findings further suggested that specific transport mechanisms are likely involved in keratin granule translocation.

### Myosin II contributes to the speed and directionality of keratin granule movement

In trying to identify likely candidate motor proteins that contribute to the directed transport of mutant keratin granules, we reasoned that microtubule-dependent motors confer typically much faster transport rates than those observed in our study and therefore focused on actin-dependent myosin motors. A prime candidate is non-muscle myosin II, which is known to support inward-directed transport processes and has been implicated in the regulation of keratin granule dynamics^21^. To this end, EYFP-K14R_125_C-expressing MCF-7 cells were treated with the myosin II-specific inhibitor para-nitroblebbistatin^22,23^. 30 images were recorded at 20 s intervals prior to drug addition and 50 images immediately afterwards at the same frequency without interruption. In this way, alterations in granule motility could be assessed in the same cells. The recordings were then subjected to the image analyses described above taking only solo tracks into account.

**Figure 7:**
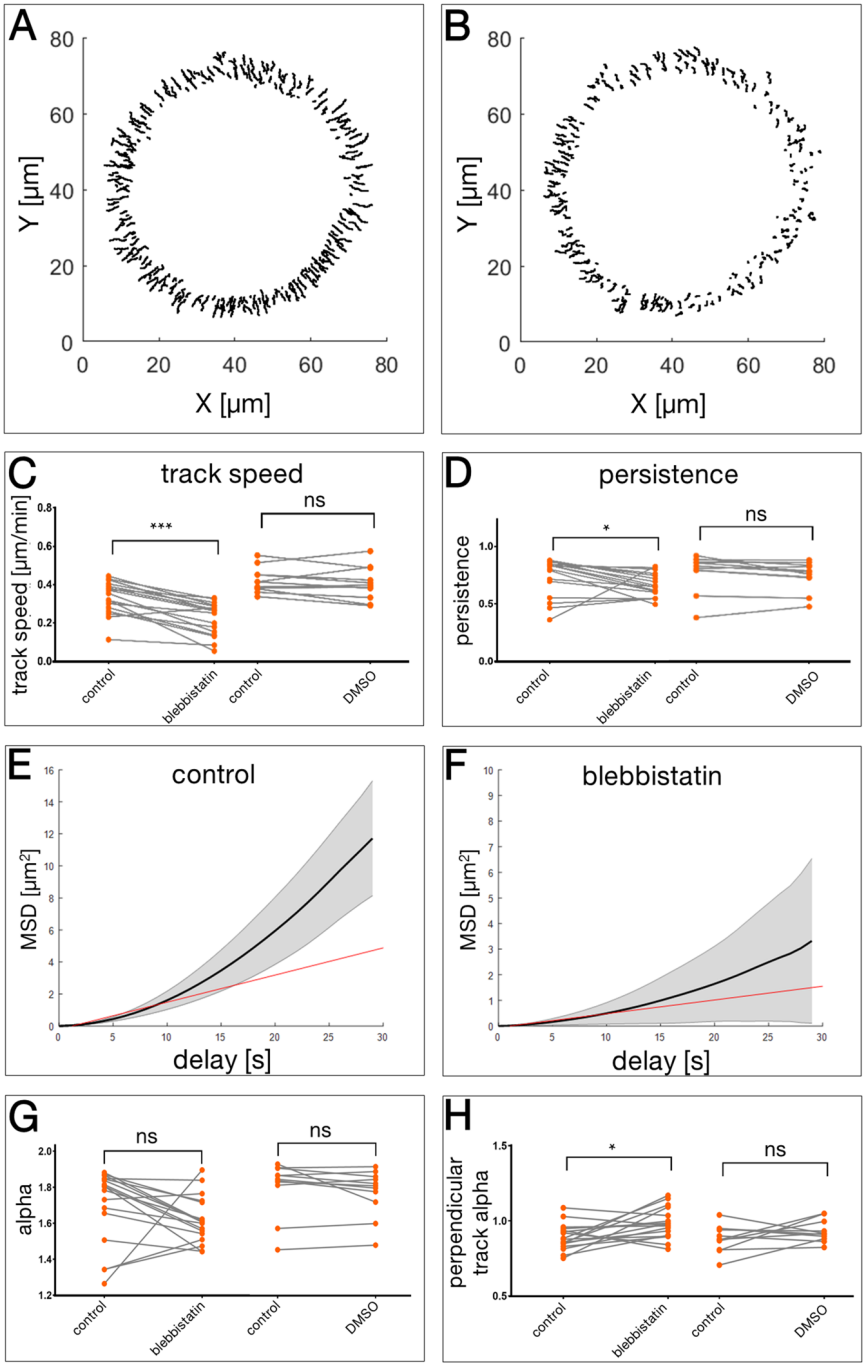
Inhibition of myosin II results in altered motility of EYFP- K14R_125_C granules. (**A**,**B**) The tracks of mutant keratin granules were retrieved from a live cell recording (20 s time intervals) using TrackMate software. The entire original time-lapse recording is provided as Movie 7. Only non-merging tracks are plotted. The tracks shown were obtained before (**A**) and after (**B**) the addition of 20 µM para-nitroblebbistatin. Note the much shorter tracks in (**B**). (**C**) The overall track speed of keratin granules before (control) and after addition of DMSO-dissolved para-nitroblebbistatin or the solvent DMSO only. For the blebbistatin experiment 17 cells were analysed yielding 900 tracks before and 989 tracks after drug addition. Note the decrease in speed from 0.332 ± 0.086 µm/min to 0.224 ± 0.086 µm/min (*p* = 0.000076). Adding DMSO does not affect speed of keratin granule movement (n = 11; 0.396 ± 0.062 µm/min vs. 0.380 ± 0.081 µm/min; *p* = 0.320). (**D**) The persistence (shortest distance between start and end point of a track divided by the actual distance travelled) of keratin granule movement is significantly reduced from 0.739 ± 0.165 to 0.664 ± 0.103 (*p* = 0.015) by para-nitroblebbistatin. Adding DMSO does not change the persistence significantly (0.729 ± 0.152 vs. 0.696 ± 0.119; *p* = 0.083). (**E**,**F**) Averaged MSD curves of the two data sets. The greyed area represents the weighted standard deviation over all MSD curves while the black line is the weighted average over all MSD curves. Fitting the first 50% of the mean curve yields the average diffusion coefficients *D* (red line) for both situations. (**G**) Differences in the alpha coefficients for the same dataset used in (**C**). Alpha = 0 indicates free Brownian motion, while values > 1 indicate directed transport, and values < 1 indicate confined motion. Adding para-nitroblebbistatin reduces alpha from 1.699 ± 0.206 to 1.619 ± 0.131 and adding DMSO reduces alpha from 1.710 ± 0.152 to 1.676 ± 0.140) both of which is statistically not significant (*p* = 0.057 and *p* = 0.102, respectively). (**H**) The "perpendicular track alpha" coefficients for the same dataset used in (**C**–**E**). Adding para-nitroblebbistatin significantly increases the average alpha from 0.890 ± 0.086 to 0.981 ± 0.102 (*p* = 0.020), the addition of DMSO does not (0.883 ± 0.090 vs. 0.931 ± 0.073; *p* = 0.320).

Blebbistatin treatment frequently led to flattening of cells and extension of cell protrusions. Solo tracks deduced from a cell before and after blebbistatin addition are shown in Fig. 7A,B, respectively (Movie 7). The decrease of track lengths after addition of para-nitroblebbistatin was obvious. The track speed changes were then analysed in 17 blebbistatin-treated cells and compared to those in 11 control cells that were only treated with the DMSO solvent. No significant changes were observed in the DMSO-treated controls whereas a highly significant decrease of mean granule speed from 0.33 to 0.22 µm/min was detected in blebbistatin-treated cells (Fig. 7C). Furthermore, the persistence, defined as the shortest distance between start and end point of a track divided by the actual distance travelled, was also significantly reduced from 0.74 to 0.66 after inhibitor addition (Fig. 7D). Averaged MSD curves of tracks in a single cell before and after addition of blebbistatin are depicted in Fig. 7E,F. Both the individual and weighted curves are considerably flattened in the presence of blebbistatin, indicating a decrease in total motility of mutant keratin granules. The alpha value calculated from the MSD analyses, however, did not change in the presence of blebbistatin or DMSO (Fig. 7G), remaining above 1 in all conditions. Similarly, the alpha value calculated along the x-axis was not altered (not shown). But the alpha along the y-axis was significantly, although only to a minor degree, increased after blebbistatin addition (Fig. 7H).

To further substantiate the inhibitor experiments, cells were treated with ML-7 as an alternative upstream myosin inhibitor (Supplementary Fig. S2). Analysis of the speed of granule movement showed a significant reduction from 0.28 to 0.17 µm/min.

Together, the observations indicated that inhibition of myosin II significantly slowed down the inward movement of mutant keratin granules.

### Movement of mutant keratin granules coincides with cortical acto-myosin flow

**Figure 8:**
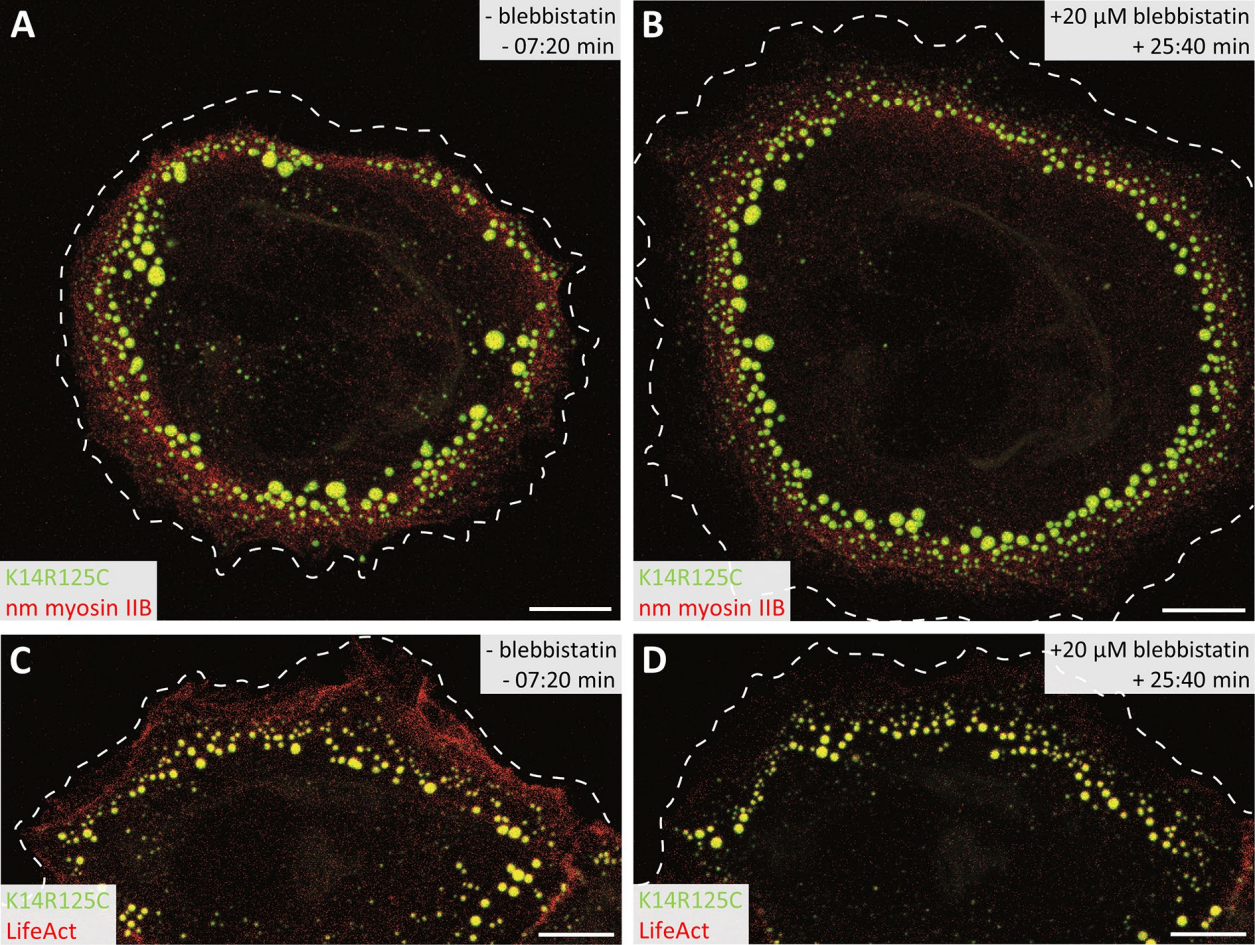
Live-cell imaging reveals interconnected retrograde movement of mutant keratin granules and cortical acto-myosin. (**A**,**B**) The projection views depict EYFP-K14R_125_C-containing keratin granules (green) and mCherry-non-muscle myosin IIB (red) in an MCF-7 cell 7 min 20 s before and 25 min 40 s after treatment with 20 µM para-nitroblebbistatin. Note the spatial proximity of the fluorescent keratin granules and dotted myosin fluorescence in the peripheral lamellum and their reduced presence in the central cytoplasm. The corresponding Movie 9 shows that both move coordinately from the cell periphery to the cell center. Note the enlargement of the cell caused by blebbistatin-induced relaxation of the cell cortex moving also keratin granules and myosin outwards. The cell membrane is demarcated by a dashed line as determined from corresponding brightfield images. Scale bars: 10 µm. (**C**,**D**) The projection views depict EYFP-K14R_125_C (green) distribution together with LifeAct-RFP (red) in an MCF-7 cell 7 min 20 s before and 25 min 40 s after treatment with 20 µM para-nitroblebbistatin. Note that cortical actin co-distributes with nascent keratin granules in the outermost region of the lamellum. The entire time-lapse sequence is presented in Movie 10. Note again the relaxation of the cell cortex and considerably reduced actin signal upon myosin II inhibition. Dashed lines indicate the cell membrane. Scale bars: 10 µm.

In view of the partial myosin-dependency of keratin granule movement, we compared motion of granules with that of myosin. To this end, MCF-7 cells stably expressing EYFP-K14R_125_C were transiently transfected with fluorescent protein-tagged non-muscle myosin IIB. The keratin granule-bearing zone coincided with the peripheral enrichment zone of non-muscle myosin IIB (Fig. 8A). Time-lapse fluorescence recording revealed that the constant myosin flow corresponded with the inward flow of keratin granules (Movies 8 and 9). Both types of motility were equally affected by para-nitroblebbistatin treatment (Fig. 8A, B; Movie 9). To understand why residual inward-directed motility persisted in the presence of blebbistatin, we examined actin dynamics using LifeAct in comparison to keratin granule mobility. In untreated cells, keratin granule motility corresponded well to the inward movement of actin often co-distributing with transverse actin arcs (Fig. 8C,D; Movie 10). As to be expected^24–26^, blebbistatin dissolved transverse arcs but did not completely abolish retrograde actin flow, which still coincided with the remaining granule movement. We therefore concluded that the inward movement of mutant keratin granules is linked to retrograde actin flow.

## Discussion

Ex vivo analyses are not sufficient to understand and characterize the mechanisms and consequences of pathological keratin aggregate formation. Thus, recombinant K14R_125_H forms prototypical intermediate filaments under optimal in vitro conditions^27^. Production of the same or similar keratin 14 mutants in cultured cells, however, leads to abundant keratin granule formation with little remaining filamentous keratins in the perinuclear region^28–31^. Time-lapse imaging revealed that these keratin granules are highly dynamic, displaying a high degree of motility and a limited lifetime. These properties offer an inroad into the investigation of factors that modulate keratin aggregation. Progress has been hampered so far by the multifactorial regulation of keratin granule dynamics. Quantitative parameters are therefore needed to determine the contribution of individual factors. Microscopic recording of fluorescence-tagged keratin mutants provides temporal and spatial information that can be dissected by image analysis to obtain such parameters. By pursuing this approach, we were able to digitally track keratin granule formation, growth, movement and disassembly at the single granule level. The results demonstrate that the determined parameters do not reflect random processes but must be the consequence of coordinated regulation and can therefore be described in quantitative terms. Specifically, we found that granule formation is restricted to less than 10 µm from the plasma membrane, that granule growth is continuous up to a certain plateau size for about 30 min, that granule mobility is consistently directed toward the cell centre, that the speed of granule movement differs between cells but always slowly declines as granules grow, and that granule disassembly is faster than assembly and is limited to a distinct transition zone between the thin cell periphery and thicker cell centre.

Probably the most remarkable property of keratin granules is their coordinated motility. It carries the signet features of an actin-dependent transport mechanism with respect to speed, directionality and continuity. In accordance, previous studies had shown that actin filament disruption by cytochalasin or latrunculin prevented keratin granule motility whereas disruption of microtubules by nocodazole did not^9^. Whether and to which degree actin-dependent motor proteins are involved in keratin granule motility, however, could not be resolved. Using the standardized imaging conditions and methods of data analysis we addressed this aspect by treating K14R_125_C-expressing cells with the myosin inhibitors para-nitroblebbistatin and ML-7. This revealed a significant reduction, though not complete inhibition of granule motility with comparatively minor effects on directionality. The findings indicated that either drug inhibition was not complete or that other transport systems contribute to keratin granule movement. Thus, kinesin-1 has also been shown to be involved in keratin motility^32^ and may act in concert with non-muscle myosin II. The kinesin-1-dependent type of motility, however, appears to be different from the restricted inward-directed motility observed for the mutant keratin granules. Furthermore, alternative scenarios may be underlying the effect of myosin II-dependent keratin granule transport: Non-muscle myosin II either directly or indirectly affects keratin granule movement. In support of the first model physical association between keratins and non-muscle myosin IIA has been documented for keratins K8/K18 and K6 with functional implications for cell migration^21,33^. A possible mechanism for the second scenario is that actin retrograde flow is the primary stimulus for inward-directed keratin granule motility. The observation that the myosin inhibitors used in this study are known to abolish transverse actin arcs but only partially (~ 50%) abolish retrograde actin flow provides such a link^24–26^. It is fully supported by the blebbistatin-induced dissolution of transverse actin arcs and the ~ 33% reduction of keratin granule speed in the present study.

A challenge in elucidating keratin aggregate formation is the influence of context-dependent modulators and the highly variable effects of different mutations. Thus, EBS-associated mutations have been shown to affect keratin network formation differently, resulting in different functional phenotypes of cell adhesion and mechanical resilience^29,34–36^. Environmental factors including mechanical stress are known to affect mutant keratin network organization and mechanotransduction^34,37,38^. This high degree of variability and the underlying multifactorial nature of keratin aggregate formation necessitate means to assess keratin networks in quantitative terms. The presented methods offer a way to screen for modifiers of keratin granule dynamics (Fig. 1B).

While our findings are relevant for dissecting mechanisms of keratin granule-forming diseases, they also improve our understanding of normal keratin network regulation and dynamics. Despite the inability of K14R_125_ mutants to support normal keratin network formation in transfected epithelial cells, keratin granule formation and dissolution share key features with the normal keratin turnover, including induction of keratin polymerization in the cell periphery and inward-directed transport accompanied by continuing polymerization^10,39^. We therefore propose that myosin-dependency of motility applies also to the observed inward transport of growing keratin filament precursors. Remarkably, keratin filament precursors are released from the transport machinery for network incorporation at a transition zone which appears to correlate with the zone, where the mutant keratins are disassembled into subunits that can be re-used for another round of assembly and disassembly^10,40^.

The developed methods will help to clarify similarities and differences to other situations of keratin granule formation, which occur for example during mitosis, apoptosis, phosphatase inhibition or increased stress^41^. More generally, it will be important to compare key features of keratin aggregate formation and turnover with other aggregates formed in other diseases involving other cytoplasmic IFPs.

## Materials and methods

### Cells

The MCF-7 cell line producing EYFP-K14R_125_C has been described^9^. Cells were grown in uncoated flasks or on glass bottom dishes (12 mm glass-diameter, thickness 1.5#; MatTek) in Dulbecco's Modified Eagle's Medium (DMEM; Sigma Aldrich) supplemented with 10% fetal calf serum (FCS; Pan Biotech) in a humidified atmosphere with 5% CO_2_ at 37 °C. Cells were passaged once a week at a ratio of 1:5–1:30 or at 1:30–1:40 for live cell imaging. For passaging, cells were washed with and subsequently incubated for 10 min in phosphate buffered saline (PBS) without Ca^2+^ or Mg^2+^ (Sigma Aldrich). They were then incubated with accutase (Sigma Aldrich) until cells detached. They were then re-suspended in trypsin neutralizer solution (Thermo Fisher), centrifuged and seeded.

### Time-lapse microscopy

MCF-7 cells were seeded in glass-bottom dishes (12 mm glass-diameter, thickness 1.5#, MatTek) two days prior to imaging and transfected one day before imaging. The cells were transfected with 5 µg of plasmid DNA and 1.5 µl Xfect polymer in a total volume of 100 µl Xfect reaction buffer (Takara) per 2 ml of cell culture medium in 35-mm diameter dishes. The LifeAct-RFP plasmid was kindly provided by Bernd Hoffmann (Forschungszentrum Jülich^42^) and the mCherry-MyosinIIB-N-18 plasmid was purchased from Addgene (#55107; kindly provided by Michael Davidson). For live-cell imaging, cells were incubated in 25 mM HEPES-buffered DMEM without phenol red (Life technologies) supplemented with 2% fetal calf serum (Pan biotech). Fluorescence recordings were performed with a laser scanning confocal microscope (LSM 710; Carl Zeiss) using Zen black 2.1 SP3 software (Carl Zeiss). The microscope was equipped with an Airyscan detector, oil immersion objective (63 × /1.40-N.A. DIC M27) and a focus-shift correction system (DefiniteFocus; all from Carl Zeiss). In order to perform live-cell imaging, the microscope incubation chamber was pre-warmed to 37 °C and humidified. For detection of EYFP the argon-ion laser was used at 514 nm and 0.3% power together with a BP 420–480 + BP 495–620 filter (Movie 7) or the argon-ion laser was used at 488 nm and 0.1–0.3% power and signal emission was acquired at a range of 505–555 nm (Movies 8, 9, 10). For detection of mCherry in living cells a 543 nm HeNe-laser (module LGK 7786 P) was used at 5.0% power and signal emission was acquired at a range of 600–700 nm. For detection of RFP a 543 nm HeNe-laser (module LGK 7786 P) was used at 10% power with signal emission being acquired at a range of 600–700 nm. The experiments were performed at an optical resolution corresponding to approximately 200–300 nm resolution in x/y length for the YFP signal. To improve accuracy, the scan-pixel-size was set to 77 nm. Microscopy images were processed in Fiji distribution of ImageJ software package^43,44^ and further processed for visualization by Adobe Photoshop CS3 and Microsoft Office Professional Plus 2016. The scheme in Fig. 1 was prepared using Adobe Illustrator CS3.

### Inhibitor treatment

Cells were seeded on day 0 on glass-bottom dishes and were imaged one day after seeding in DMEM including 4-(2-hydroxyethyl)-1-piperazineethanesulfonic acid (HEPES) and without phenol red (Sigma Aldrich) supplemented with 2% FCS (Pan Biotech). Live cell imaging was performed on a Zeiss LSM710 Duo microscope at 37 °C. The 488 nm line of an argon/krypton laser was used for fluorescence recording with a 63 × /1.40-N.A. DIC M27 oil immersion objective. The emitted light was monitored between 500 and 540 nm (green signal), with a pinhole set at 1–2 AU (airy unit) and a laser intensity of 0.2%. Cells were imaged in control medium. Subsequently, the medium was aspirated using a vacuum pump and replaced by medium supplemented with the respective inhibitor for further imaging without interruption of recording. Para-nitroblebbistatin was purchased from Optopharma. Concentrations of 5, 10, 20 and 50 µM para-nitroblebbistatin were tested. While 5 µM inhibited keratin granule motility only mildly, 50 µM showed signs of toxicity, completely inhibiting intracellular motility and dynamics. Based on the test results and the recommendation of the manufacturer to not exceed 20 µM because of unspecific side effects, the reported efficient inhibition of myosin 2 function in cultured cells using 10–20 µM para-nitroblebbistatin^22^, the limited solubility of blebbistatin (4–25 µM in aqueous solution containing 0.1–2% DMSO for blebbistatin^22,23^), and the concern for toxicity of high para-nitroblebbistatin concentrations we decided to use 20 µM as the standard concentration.

ML-7 was purchased from Tocris and used at a standard concentration of 20 µM which has been shown to inhibit MLCK efficiently in MCF-7 cells and is known to avoid unspecific inhibition of protein kinase A and C^45,46^.

### Automated image analysis

For best comparison high quality recordings of isolated cells were selected that had a comparable overall cell morphology (round shape, flat cell body). Image sequences were either recorded over a long period (between 270 and 365 frames per movie with 3 frames/min) allowing the analysis of complete tracks, or in a before–after fashion to study the impact of modulators. In both instances, the analysis followed the scheme shown in Fig. 1B. First, granules were tracked with the help of the FIJI plugin TrackMate^47^. For segmentation, the LoG detector was applied with the following parameters: blob diameter = 1 µm; threshold = 33 (in some experiments 1, due to different recording settings); no mean filtering; and sub-pixel localization active. For tracking, the LAP tracker was used with the following parameters: Frame to frame linking = 1 µm; track segment gap closing with maximal distance of 1 µm and maximal frame gap of 2; track segment splitting disabled; track segment merging 1 µm. After initial track calculation, the tracks were filtered for mean quality set to "auto". For further inspection numerical data (track-IDs; x, y, t; fluorescence) were exported and MATLAB (The MathWorks, Inc.) custom scripts were used. Since merging of tracks occurred regularly, tracks were automatically classified as solo tracks which did not merge, and merging tracks that were subdivided into main tracks and sub tracks (Supplementary Fig. S1A). Mean local track speed, local advance speed and fluorescence of granules were calculated for the different track types. To consider local changes of speed, a gliding mean of speed was calculated by computing the local track and advance speeds for each time point taking 8 image recordings from t − 3 to t + 4 into account (Fig. S1B).

To further examine the motion properties of granules, MSD analysis was performed using the MATLAB class @msdanalyzer^48^. The coefficient alpha was determined in the following way: The MSD function was calculated for each individual trajectory and its log–log representation was fitted with a linear function such that if the MSD curve could be modelled by ρ(r) = ⟨r2⟩ = Γta, then log(⟨r2⟩) = f(log(t)) was fitted with Γ + α log(t). We fitted individual MSD curves and discarded those for which the R2 coefficient, reflecting the quality of the fit, was < 0.8^48^. For MSD analysis along or perpendicular to the track direction, tracks were rotated in a way that start- and end-point were located horizontally. In that instance, x values reflect back and forth movements while y-values reflect up and down movements, i.e. movements perpendicular to the track axis. MSD analysis then was performed for x and y values separately.

To test significance of before and after parameters the signrank function applying the Wilcoxon matched-pairs signed-ranks test^49^ was used. Graphs were prepared using GraphPadPrism v7 Software. Values are given with standard deviation of the mean, i.e. ± SD. Significance with a *p* ≤ 0.001 was labelled ***, *p* ≤ 0.01 was labelled ** and *p* ≤ 0.05 was labelled *.

## Supplementary Information

Supplementary Movie 1

Supplementary Movie 2

Supplementary Movie 3

Supplementary Movie 4

Supplementary Movie 5

Supplementary Movie 6

Supplementary Movie 7

Supplementary Movie 8

Supplementary Movie 9

Supplementary Movie 10

Supplementary Information

## Data Availability

The automated image analysis protocols generated during the current study are available in the GitHub repository, https://github.com/rwindoffer/KER_TRACK.
