# Supplementary material for: Growth, lifetime, directional movement and myosin-dependent motility of mutant keratin granules in cultured cells
Source: Sci Rep. 2021 Jan 27;11:2379. doi: 10.1038/s41598-021-81542-8 (PMC7840912; doi:10.1038/s41598-021-81542-8)
Supplement: Supplementary file 11 — Supplementary Information [file 41598_2021_81542_MOESM11_ESM.docx]

**Growth, lifetime, directional movement and myosin-dependent motility of mutant keratin granules in cultured cells**

S. M. Lehmann, R. E. Leube, R. Windoffer

**Supporting information**


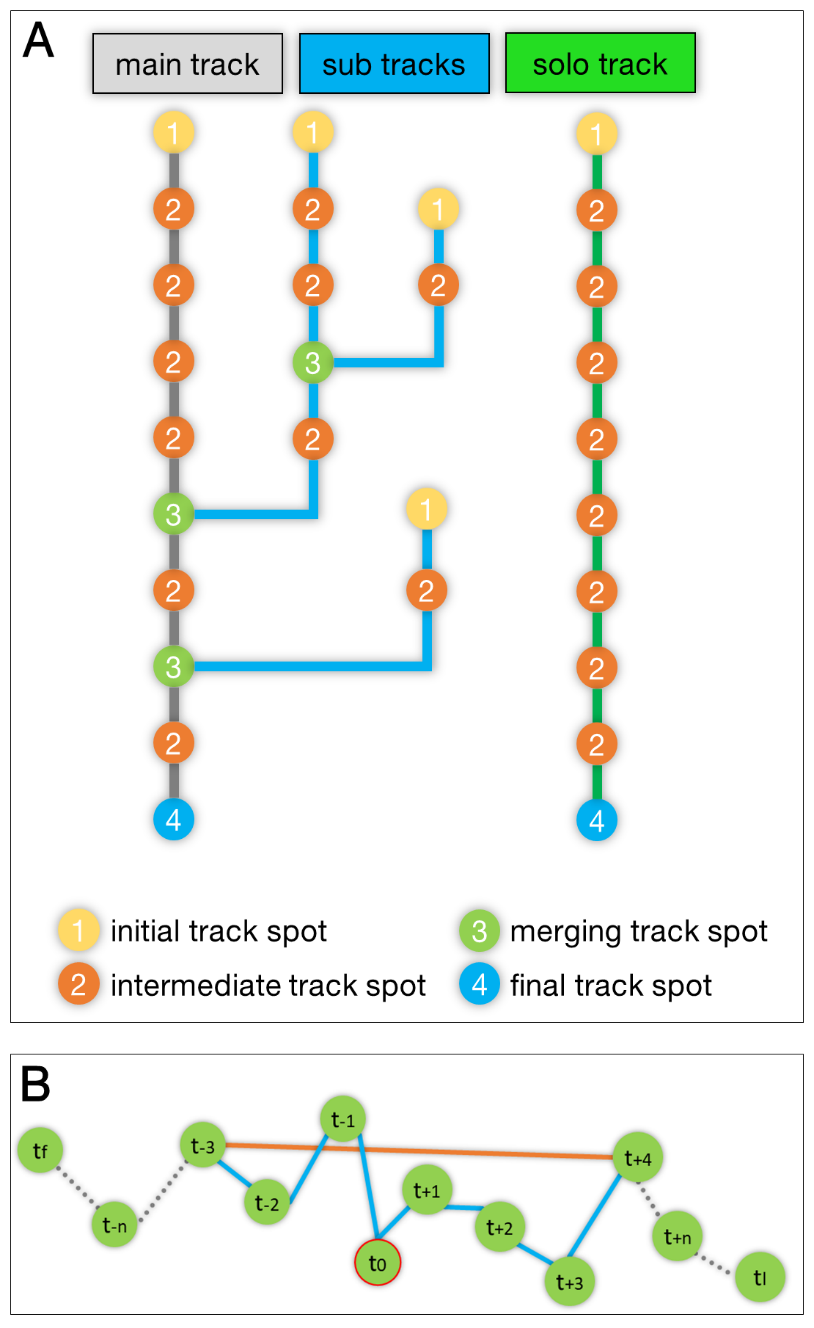


**Figure S1. Schematic overview of track analyses.**

A illustrates the categorization of track subtypes. Solo tracks represent granules, which do not fuse with any other trackable granule. Granules, which merge with each other, are separated into sub tracks, with the longest sub track being referred to as the main track. Positions of granules are distinguished as the initial track spot position (1), where granules can be first detected, the final track spot position (4), where granules are last detected, and intermediate track spot positions corresponding to the in between positions (2). In addition, a merging track spot position (3) is defined as the position at the time point when two merging granules are detected as one spot.

B Schematic example for the calculation of local track and advance speed at time point t_0_. Positions of granules are shown as green circles at different time points. Local track speed is defined as the track length (blue line) from t_-3_ to t_+4_ divided by time (t_+4_ – t_-3_). Local advance speed is defined as the distance (orange line) covered between t_-3_ and t_+4_ divided by the evolved time. Local speed values are calculated for other time points in the same manner.

**
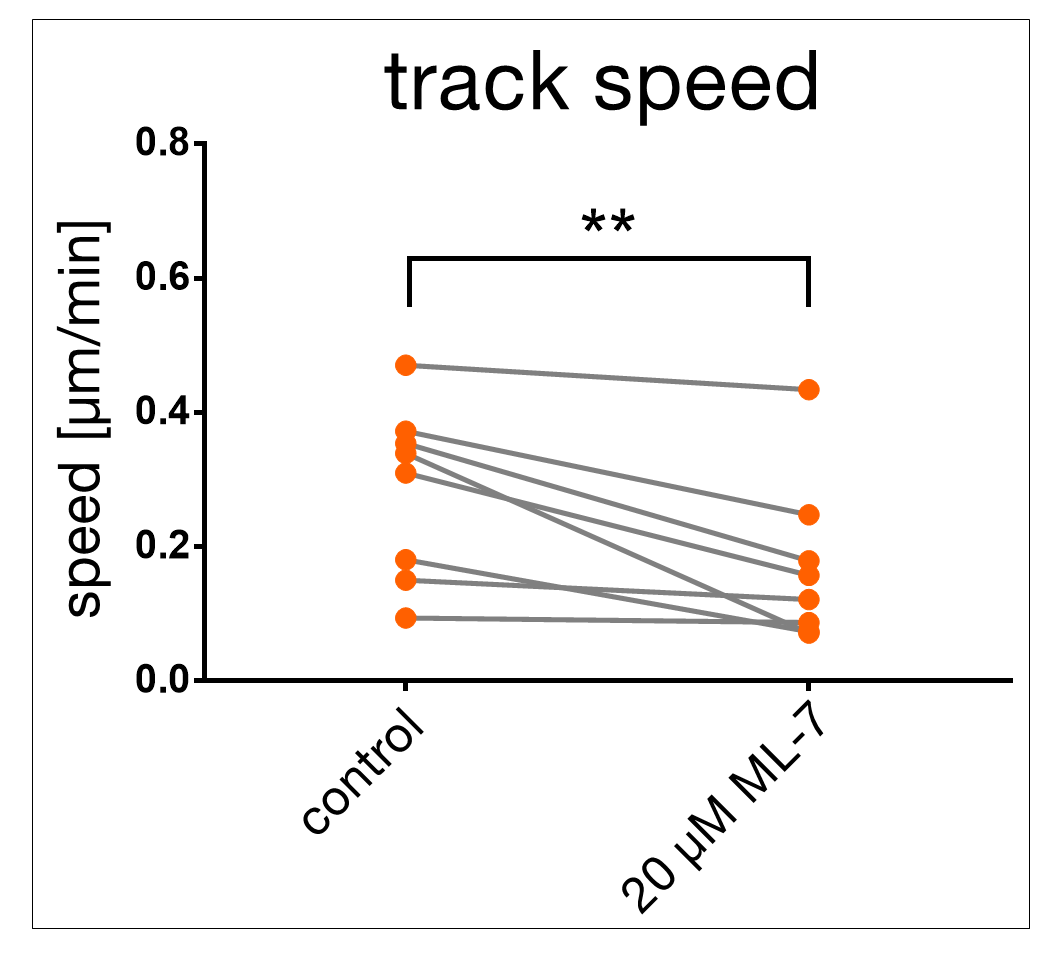
**

**Figure S2. Inhibition of non-muscle myosin II by ML-7 reduces the track speed of mutant keratin granules.**

The graph depicts the track speed of keratin granules before (control) and after addition of 20 µM ML-7, a selective myosin light-chain kinase inhibitor. 8 cells were analysed yielding 616 tracks before and 929 tracks after drug addition. The decrease in overall speed from 0.284±0.129 µm/min to 0.171±0.122 µm/min is significant (p=0.008).

**Movie Legends**

**Movie 1.** Time-lapse fluorescence recording of EYFP-K14R_125_C in an MCF-7 cell. Shown is the inverse projection view of 5 focal planes, covering most of the keratin granule signal. Recording frequency: 3 frames/min. Corresponding Fig. 2A shows the still image recorded at time point 0 min.

**Movie 2.** (A) Image analyses of the time-lapse fluorescence recording of EYFP-K14R_125_C in the MCF-7 cell depicted in Movie 1. The four quadrants illustrate different stages of image documentation and analysis as described in corresponding Fig. 2B.

(B) The segment is a magnification of (A) (see boxed area in Fig. 2B) and shows the original recordings, the derived tracks of detected granules (magenta circles), the number of granule merging events, and the speed of keratin granules along individual tracks for each time point. Further information is provided in the legend to corresponding Fig. 2C.

**Movie 3.** Image analyses of time-lapse fluorescence recording of EYFP-K14R_125_C in an MCF-7 cell (recording frequency: 3 frames/min). The track of a single, non-fusing granule is highlighted in green (see also corresponding Fig. 2D). Magenta circles highlight granules from other tracks.

**Movie 4.** Image analyses of time-lapse fluorescence recording of EYFP-K14R_125_C in an MCF-7 cell (recording frequency: 3 frames/min). The tracks of three fusing granules are demarcated in green (see also corresponding Fig. 2E). Magenta circles show granules from other tracks.

**Movie 5.** Analysis of changing fluorescence (brightness) in a single granule that is tracked in a time-lapse fluorescence recording of EYFP-K14R_125_C in an MCF-7 cell (corresponding Movie 3). The fluorescence intensity was measured in a single, non-fusing granule moving along the track highlighted in magenta at top (see also corresponding Fig. 4C).

**Movie 6.** (A) Analysis of changing local track speed, (B) analysis of changing local advance speed, and (C) analysis of changing local advance speed to local track speed ratios of a single granule that is monitored in a time-lapse fluorescence recording of EYFP-K14R_125_C in an MCF-7 cell (corresponding Movie 3). The values were determined for a single, non-fusing granule moving along the track highlighted in magenta at top (see also corresponding Fig. 5M-0).

**Movie 7.** Fluorescence recording of EYFP-K14R_125_C in an MCF-7 cell. Shown are the inverse projection views of 6 focal planes with a total height of 3.44 µm. The cell was incubated in normal medium for 10 min and medium was subsequently replaced with medium containing 20 µM para-nitroblebbistatin until 26 min 20 s (see also corresponding Fig. 7A,B).

**Movie 8.** Fluorescence recording of EYFP-K14R_125_C and mCherry-non-muscle myosin IIB in an MCF-7 cell. Projection view of 3 focal planes with a total height of 1.0 µm for 4 min 45 s.

**Movie 9.** Fluorescence recording of EYFP-K14R_125_C mCherry-non-muscle myosin IIB in an MCF-7 cell. Projection view of 4 focal planes with a total height of 1.5 µm. Recording frequency: 3 frames/min. The cell was incubated in normal medium for 7 min and subsequently medium was replaced with medium containing 20 µM para-nitroblebbistatin and imaged for another 25 min 40 s. The keratin track speed was reduced by 24.6% in the treated cell (see also corresponding Fig. 8A,B).

**Movie 10.** Fluorescence recording of EYFP-K14R_125_C and LifeAct-RFP in an MCF-7 cell. Projection view of 4 focal planes with a total height of 1.5 µm. Recording frequency: 3 frames/min. The cell was incubated in normal medium for 7 min and subsequently medium was replaced with medium containing 20 µM para-nitroblebbistatin and imaged for another 25 min 40 s. Keratin granule track speed was reduced by 27.8% in the treated cell (see also corresponding Fig. 8C,D).
